# Genetics and fine mapping of a purple leaf gene, *BoPr*, in ornamental kale (*Brassica oleracea* L. var. *acephala*)

**DOI:** 10.1186/s12864-017-3613-x

**Published:** 2017-03-14

**Authors:** Xiao-ping Liu, Bao-zhen Gao, Feng-qing Han, Zhi-yuan Fang, Li-mei Yang, Mu Zhuang, Hong-hao Lv, Yu-mei Liu, Zhan-sheng Li, Cheng-cheng Cai, Hai-long Yu, Zhi-yuan Li, Yang-yong Zhang

**Affiliations:** Institute of Vegetables and Flowers, Chinese Academy of Agricultural Sciences, 12# ZhongGuanCun Nandajie, 100081 Beijing, People’s Republic of China

**Keywords:** Ornamental kale, Purple leaf, Inheritance, Fine mapping, Candidate gene

## Abstract

**Background:**

Due to its variegated and colorful leaves, ornamental kale (*Brassica oleracea* L. var. *acephala*) has become a popular ornamental plant. In this study, we report the fine mapping and analysis of a candidate purple leaf gene using a backcross population and an F_2_ population derived from two parental lines: W1827 (with white leaves) and P1835 (with purple leaves).

**Results:**

Genetic analysis indicated that the purple leaf trait is controlled by a single dominant gene, which we named *BoPr*. Using markers developed based on the reference genome ‘02–12’, the *BoPr* gene was preliminarily mapped to a 280-kb interval of chromosome C09, with flanking markers M17 and BoID4714 at genetic distances of 4.3 cM and 1.5 cM, respectively. The recombination rate within this interval is almost 12 times higher than the usual level, which could be caused by assembly error for reference genome ‘02–12’ at this interval. Primers were designed based on ‘TO1000’, another *B. oleracea* reference genome. Among the newly designed InDel markers, BRID485 and BRID490 were found to be the closest to *BoPr*, flanking the gene at genetic distances of 0.1 cM and 0.2 cM, respectively; the interval between the two markers is 44.8 kb (reference genome ‘TO1000’). Seven annotated genes are located within the 44.8 kb genomic region, of which only *Bo9g058630* shows high homology to AT5G42800 (dihydroflavonol reductase), which was identified as a candidate gene for *BoPr*. Blast analysis revealed that this 44.8 kb interval is located on an unanchored scaffold (Scaffold000035_P2) of ‘02–12’, confirming the existence of assembly error at the interval between M17 and BoID4714 for reference genome ‘02–12’.

**Conclusions:**

This study identified a candidate gene for *BoPr* and lays a foundation for the cloning and functional analysis of this gene.

**Electronic supplementary material:**

The online version of this article (doi:10.1186/s12864-017-3613-x) contains supplementary material, which is available to authorized users.

## Background

Ornamental kale (Brassica *oleracea* L. var. *acephala*) is a popular ornamental plant cultivated worldwide owing to its variegated, colorful leaves [1] and strong resistance to cold. The leaves of ornamental kale are diverse and colorful: the edges are emerald green, dark-green, gray-green, or yellow-green, and the center can be white, yellow, pink, red, purple or other complex color varieties. The leaf pattern of ornamental kale is classified as foliage leaves, round leaves, cracked leaves, wave leaves and other types. Furthermore, purple-leaf ornamental kale, which is mainly due to various anthocyanin components [2], is reported to possess strong antioxidant capacity and thus potential benefits to human health [3, 4].

Some leaf color genes have been mapped in *Brassica* crops. For example, the red leaf color trait is reportedly controlled by the single dominant gene *Re* in ornamental kale, which was mapped to C09 between SSR markers C9Z90 and C9Z94, with genetic distances of 0.3 cM and 2.0 cM, respectively [5]. In *Brassica juncea,* the purple leaf gene (PL) was mapped between SRAP markers ME7EM9 and ME2EM2, with genetic distances of 1.2 cM and 5.5 cM, respectively [6]. A purple leaf dominant gene (*BrPur*) in *Brassica rapa* was assigned to a locus between InDel markers BVRCPI613 and BVRCPI431, with a genetic interval of 0.6 cM [7]. An incomplete dominant gene (*BnaA.PL1*) for the purple leaf trait in *Brassica napus* has been mapped, and the possible candidate gene is predicted to encode adenosine 5’-phosphosulfate reductase [8]. Zhu et al. found the pink leaf color trait in ornamental kale to be controlled by a single semi-dominant gene mapped to C03 between SSR marker Ni2C12 and co-dominant SCAR marker Boac04, with genetic distances of 0.6 cM and 2.4 cM, respectively [9].

With the development of molecular biology and the release of the *B. oleracea* draft genome [10, 11], uncovering the genetic basis of important traits has become a focus of theoretical and applied studies in this species. To date, many genes/QTLs have been mapped in *Brassica oleracea* using InDel markers, including the Fusarium wilt resistance gene *FOC1* [12, 13], the petal color gene *cpc-1* [14], the yellow-green gene *ygl-1* [15], and QTLs associated with heading traits [16], head-splitting resistance [17, 18], Diamondback (*Plutella xylostella*) moth resistance [19], and resistance to diseases such as *Sclerotinia sclerotiorum* [20], black rot [21, 22], and clubroot [23].

In this study, kale inbred line W1827 (with white leaves) was crossed to inbred line P1835 (with purple leaves) to construct F_2_ and backcross (BC) populations, which were used for genetic analysis and fine mapping of the gene for the purple leaf trait. Genes located within the fine-mapping region were analyzed. These findings will lay a foundation for the cloning and validation of *BoPr* and will facilitate elucidation of the molecular mechanism for purple leaf formation in *B. oleracea* L. var. *acephala*.

## Methods

### Plant materials

**Fig. 1:**
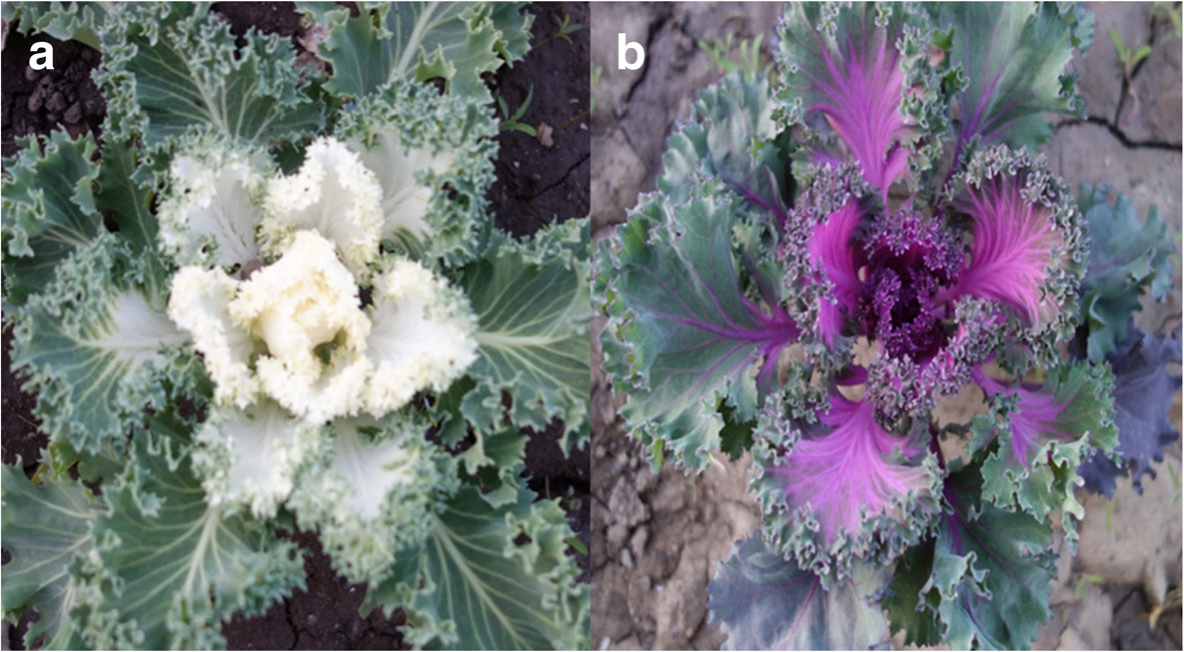
Phenotypes of the parental lines. **a** W1827. **b** P1835

The female kale inbred line W1827 (P_1_, white leaf, Fig. 1a) was crossed with male kale inbred line P1835 (P_2_, purple color, Fig. 1b) to generate the F_1_ population. The F_2_ population was obtained via self-pollination of F_1_ plants; BC_1_P_1_ and BC_1_P_2_ populations were created by backcrossing of F_1_ × W1827 and F_1_ × P1835, respectively. All materials were from Cabbage and Broccoli Research Group, Institute of Vegetables and Flowers (IVF), Chinese Academy of Agricultural Sciences (CAAS).

### Genetic analysis and InDel marker development

Leaf color was identified visually. The segregation ratios of the F_2_ and BC_1_ populations were analyzed using a Chi-square test with SAS software.

The ‘02–12’ reference genome of cabbage was downloaded from BRAD (http://brassicadb.org) [10]; the TO1000 sequence was obtained from the genome sequence (http://plants.ensembl.org/Brassica_oleracea) [11]. The sequencing-by-synthesis method was used to re-sequence the parents at approximately 10× coverage over the entire genome [24]. This work was completed at Beijing Genomics Institute (BGI) (Shenzhen, China). For preliminary mapping, the re-sequencing data for both parents was mapped to the ‘02–12’ reference genome of *B. oleracea* (http://brassicadb.org) to detect 3-5 bp insertion-deletion mutations between the parents. InDel primers were then designed. Due to the possible assembly error of the 02–12 reference genome, new primers in the preliminary mapping region were designed based on the TO1000 reference genome. Primers were designed to have amplicon lengths of 100–200 bp, GC contents of 40–50% and Tm values of 52–62 °C.

### DNA extraction and bulked segregant analysis (BSA)

Genomic DNA was extracted from fresh leaves using a modified cetyltrimethylammonium bromide (CTAB) protocol [25]. The concentration of DNA was estimated using a spectrophotometer (BioDrop, UK) and diluted to 40–50 ng/μL.

Ten purple-leaf F_2_ individuals and ten white-leaf F_2_ individuals were selected to construct two pools using the BSA method [26]. Polymorphic InDel markers between the parents were used to screen these pools, and polymorphic markers between the pools were used to examine recombination with all white-leaf individuals in the F_2_ and BC_1_P_1_ populations.

The 20 μL PCR reaction mixture contained 4 μL DNA template (40–50 ng/μL), 2 μL 10× PCR buffer (Mg^2+^ included), 1.6 μL dNTPs (2.5 mM each), 0.8 μL forward primer (10 μM), 0.8 μL reverse primer (10 μM), 0.4 μL Taq DNA polymerase (2.5 U/μL) and 10.4 μL ddH_2_O. Reactions were performed in a thermal cycler as follows: 94 °C for 5 min; 35 cycles of 94 °C for 30 s, 55 °C for 30 s and 72 °C for 45 s; 72 °C for 10 min. Amplicons were separated by 8% PAGE at 160 V for 1.5 h, followed by silver staining.

### Data analysis

For each marker, individuals with the W1827 allele were categorized as ‘a’, and individuals with the P1835 allele were categorized as ‘b’; those with the F_1_ allele were categorized as ‘h’. The Kosambi mapping function was used to calculate genetic distances between markers [27], and the genetic map was constructed using MapDraw [28].

To identify probable genes associated with purple leaves, genes located within the candidate interval were analyzed based on annotations from the *B. oleracea* reference genome ‘TO1000’.

### Analysis of the candidate gene

To amplify the candidate gene Bo9g058630, primers were designed in DNAMAN 7.0 using the sequence of Bo9g058630. PCR amplification was performed using the Q5 Ultra High Fidelity DNA polymerase. The PCR amplification conditions followed the manufacturer’s specifications. The annealing temperature was determined using the NEB Tm calculator (New England Biolabs, Inc.), and the elongation step was based on a rate of 30 s/kb. The candidate gene sequence was determined by Sanger sequencing of the PCR amplicons.

## Results

### Genetic analysis of leaf color of W1827 with P1835

Sixteen F_1_ individuals generated by crossing W1827 with P1835 exhibited purple leaves, indicating that the purple leaf color trait is dominant over the white-leaf trait. In the F_2_ population, 258 of 1008 individuals displayed white leaves and 750 purple leaves. The Chi-square test confirmed this segregation ratio to be 3:1. In the BC_1_P_1_ population, 1040 of 2034 individuals showed purple leaves and 994 white leaves, with the Chi-square test confirming the segregation ratio to be 1:1. The leaves of all 200 individuals in the BC_1_P_2_ population were purple (Table 1). These results indicated that the purple leaf trait is controlled by a single dominant gene, which we termed *BoPr*.

**Table 1:** The Chi-square (*χ* ^2^) goodness-of-fit test ratios of leaf color segregation in BC and F_2_ populations

Populations	Total plants number	Number of purple leaf individuals*	Number of white leaf individuals	Expected ratio	*χ* ^2a^
F_1_	16	16	0	-	-
F_2_	1008	750	258	3:1	0.19
BC_1_P_1_	2034	1040	994	1:1	0.52
BC_1_P_2_	200	200	0	-	-

*Purple plants and white plants were determined by visual inspection

^a^ *χ* ^2^ > χ^2^_0.05_ = 3.84 was considered significant

### Preliminary mapping of *BoPr*

Illumina paired-end sequencing generated reads of 9.2 Gb for kale inbred line W1827 and 9.9 Gb for line P1835. A total of 211 pairs of InDel marker primers were designed by comparing the whole-genome re-sequencing data with the sequence of the ‘02–12’ reference genome.

These InDel marker primers were used to screen for polymorphisms between the parents. Ultimately, 58 of the 211 pairs of primers revealed polymorphisms, with a polymorphic rate of 27.5%. The polymorphisms were further screened in two the bulks of the F_2_ population, and only BoID4814, BoID4826 and BoID4837 exhibited polymorphisms between the two bulks. The three markers were preliminarily located on chromosome C09 according to the marker location in the reference genome.

**Fig. 2:**
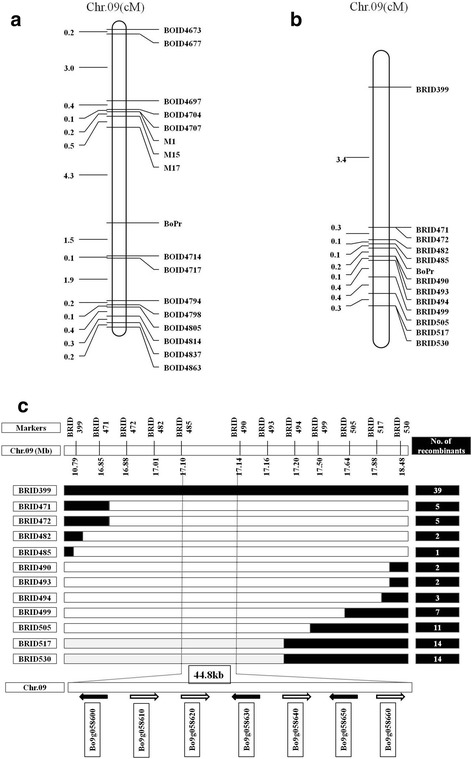
**a** Preliminary mapping of *BoPr*, units: cM. **b** Fine mapping of *BoPr*, units: cM. **c** Fine mapping using the BC_1_P_1_ population with 2034 plants. The *BoPr* gene was delimited to an interval between BRID485 and BRID490, with an estimated length of 44.8 kb, seven genes were annotated between markers BRID485 and BRID490 based on the reference genome sequence. The genetic structure for each recombinant type is depicted as white for homozygous white leaf alleles, black rectangles for heterozygous alleles, respectively. The relative positions of markers on C09 were determined according to the TO1000 genome sequence

Subsequently, 41 new additional InDel markers near the three polymorphic primers were designed, 26 of which showed polymorphisms. Only co-dominant markers with clear and stable amplification were chosen for further analysis in the F_2_ and BC_1_P_1_ populations. A genetic map comprising 16 InDel markers (Table 2) was constructed using MapDraw [26] (Fig. 2a). InDel markers M17 and BoID4714 were found to be closest to *BoPr*, flanking the gene at genetic distances of 4.3 cM and 1.5 cM, respectively. Based on marker locations in the reference genome ‘02–12’, *BoPr* is located on chromosome C09. The interval between the two markers is 280 kb (6,312,350–6,592,994 bp).

**Table 2:** The InDel primer sequences used in this study

Primer name	Physical distance (bp) (02-12)	Forward primer sequence (5’-3’)	Reverse primer sequence (3’-5’)
BoID4673	C09:4123400	GAATCGAGACAGAACCGTAT	TAACGTTAACCGGATTGG
BoID4677	C09:4347473	TTAGCGTTTGTGTATTACCG	AAAGAACACAGAGGTTTGGA
BoID4697	C09:5350367	GGCTTTCTATCTGTCAAAAGG	GTTGGAGATTTTCTATCCGA
BoID4704	C09:5830712	TAATATTTGCGAGTGGAAGC	ATCCAGAACCGTCAATCTAA
BoID4707	C09:5940678	GTTTCACCCTTTGATCCTTT	TTCGCACCTTATCAAGTAGAG
M1	C09:5943643	TTGAGCTGCTTCCTTTAGTC	ACAGGAGTGGCATTTACATC
M15	C09:6139366	CTTACACGAACCTTCTCTCG	GGCCTGCATATAAACCTCTT
M17	C09:6312350	GAGGAGTCTACATGCGTTTC	TTGAGGGTAAAGTAGACGGA
BoID4714	C09:6592994	GCCTTAGCATCCAGAGATTA	ACGTCACAACGCTAATTACA
BoID4717	C09:7100587	TCCTGGATACGAAGTACCTG	TCCTAGAAACTTGTTGTCGC
BoID4794	C09:13019067	CATACTTGGAAGGAGCTTTG	CTCACAGTTTCTCCTTTTGG
BoID4798	C09:13119313	CGGATTAACACAGTGAAGAAG	AGATTTTGACCTTAGAAGCG
BoID4805	C09:13318893	ATATCGTCTGGCCCTCTATT	AGAACCCACAGAGACATCAC
BoID4814	C09:14085188	CCTTGGGATTTACAAGGTACT	GAGTAATCGAGAATTATGAGCC
BoID4837	C09:18142750	TGATACCTTTTGTCGAGCTT	ATTTGGGGTTGGTAGAAATC
BoID4863	C09:20025875	AGCAGTGCATGATACAAATG	CGGGCTGTCTAAATCATTA
Primer name	Physical distance (bp) (TO1000)	Forward primer sequence (5’-3’)	Reverse primer sequence (3’-5’)
BRID399	C09:10788227	GGTTGGATTTAGATTTGCTG	CTCCACCGTCATTGTATCAT
BRID471	C09:16850910	TCTAAAAGGAGAAGCCAGAA	GAGGGGAGATGAGGTTTACT
BRID472	C09:16879357	GTTTGAGTTTGTTTTGGGAG	GACTTGTAGCATTTGTCGTG
BRID482	C09:17010450	CATGCATTGAAAGTGTTGTC	AACTGAGCTTTCACACTGGT
BRID485	C09:17102497	CTTCTTGGAGGTCTCTTGTG	TGCACATTAAAACGGTAGG
BRID490	C09:17147250	TACTTGCATCAAATGTTGGG	GCACAGACTCATCTCCTTTC
BRID493	C09:17167025	AGCACTGAGACTACCCTTGA	CAGCTTTGTCTTGTGTCTGA
BRID494	C09:17205726	CGCCATATGAATAAAGGAAC	ACGAAGTCAACAAACAGCTT
BRID499	C09:17500985	ATGCGATGATGAGATAGCTT	AGATCCACTTCAATCCAATG
BRID505	C09:17643567	AACACATATTCCCGTCAAAC	GGAAAAGTGTGTTTTTGGG
BRID517	C09:17879063	AAATCATCCAAACCACTGTC	GTGCCTCTACTAGTTTTGGG
BRID530	C09:18485633	TTCAAGACTCTCCAGCTCAT	CAAGAGAAAGACTGGTTCGT

### Fine mapping of the purple leaf gene

The 280 kb interval spans two scaffolds (Scaffold000195_P2, Scaffold000050), and no polymorphic marker between the parents was found in this narrow interval. Moreover, the genetic distance for this 280 kb interval was calculated to be 5.8 cM, 12 times higher than the usual level (~600 kb/cM). Thus, another reference genome, ‘TO1000’ [11], was used to determine the accuracy of this interval. Blast alignment analysis showed the physical distance between markers M17 (7,590,215–7,590,234 bp) and BoID4714 (20,327,191–20,327,211 bp) to be approximately 12.73 Mb in the ‘TO1000’ reference genome, indicating possible assembly errors in the reference genome ‘02–12’ or ‘TO1000’. To determine whether this 12.73 Mb interval is linked to the *BoPr* gene, 70 pairs of InDel marker primers were designed based on the ‘TO1000’ reference genome. Polymorphisms between the two bulks were found for 12 of the 70 pairs, and these were used to analyze the BC_1_P_1_ and F_2_ populations. A genetic map comprising 12 InDel markers (Table 2) was constructed using MapDraw (Fig. 2b). InDel markers BRID485 and BRID490 were found to be the closest to *BoPr*, flanking the gene at genetic distances of 0.10 cM and 0.20 cM, respectively. Based on marker locations in the reference genome ‘TO1000’, the interval between BRID485 and BRID490 is 44.8 kb (17,102,497–17,147,250 bp).

### Prediction and analysis of the candidate gene

Based on the ‘TO1000’ reference genome (http://plants.ensembl.org/Brassica_oleracea) (Parkin et al. 2014), seven genes were identified in the 44.8 kb region (Table 3). According to domain annotations from InterPro and BLASTX (best hit) analyses, four of these seven genes have not been annotated (Table 3). The other three genes are as follows: Bo9g058600 (homologous gene AT5G42790) encoding the largest proteasomal subunit; Bo9g058660 (homologous gene AT5G42810) encoding inositol tetra-pentaphosphate 2-kinase; *Bo9g058630* (homologous gene AT5G42800) encoding dihydroflavonol 4-reductase (DFR), which catalyzes conversion of dihydroquercetin to leucocyanidin in anthocyanin biosynthesis [29]. Thus, we tentatively designated *Bo9g058630* as the candidate gene controlling the purple leaf color in ornamental kale.

**Table 3:** Annotation of *B. oleracea* genes in the candidate region

Bol ID^a^	E-value	Bo genes^b^	Gene position on C09^c^	AT ID^d^	E-value	AT GO^e^ annotation
Bol035270	0.0	Bo9g058600	17102783–17104636 bp	AT5G42790	0.0	the largest subunit of proteasome
-	-	Bo9g058610	17111283–17112572 bp	-	-	-
-	-	Bo9g058620	17113341–17114372 bp	-	-	-
Bol035269	0.0	Bo9g058630	17116312–17117891 bp	AT5G42800	0.0	dihydroflavonol reductase
-	-	Bo9g058640	17124223–17124381 bp	-	-	-
-	-	Bo9g058650	17137677–17138809 bp	-	-	-
Bol035268	0.0	Bo9g058660	17145032–17147070 bp	AT5G42810	0.0	Encodes an inositol tetra-/pentaphosphate 2-kinase

^a^ *B. oleracea* homologous genes in the candidate region (reference genome ‘02–12’)

^b^Seven *B. oleracea* genes in the candidate region. The likely leaf color genes and their information are indicated in bold (reference genome ‘TO1000’)

^c^The physical position of seven *B. oleracea* genes on chromosome C09 (reference genome ‘TO1000’)

^d^The best hits of the seven *B. oleracea* genes compared to *A. thaliana* (AT)

^e^GO annotations for seven Bo to AT best-hit genes obtained from TAIR

**Fig. 3:**
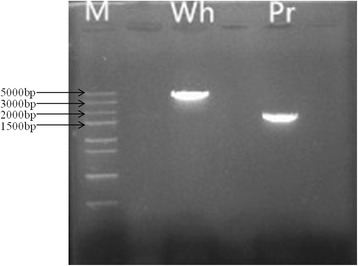
PCR products of amplicon Bo630 (isolated by agarose gel electrophoresis); M represents the DNA ladder, Pr is the inbred line P1835 with purple leaves, Wh is the inbred line W1827 with white leaves

Primer pairs were designed spanning the full length of *Bo9g058630* (Table 4), and PCR was performed using genomic DNA of W1827 and P1835 as the template. Based on agarose gel electrophoresis (Fig. 3), amplicons of approximately 4.0 kb for W1827 and approximately 2.0 kb for P1835 were obtained. Sequencing revealed that the full-length *Bo9g058630* sequence in purple-leaf P1835 is 1580 bp, whereas that in white-leaf W1827 is 3856 bp. Compared with *Bo9g058630* in P1835, a 124 bp deletion, a 2400 bp insertion at nucleotide 68 and four SNPs are present in W1827 (sequences of W1827 and P1835 are supplied in Additional file 1: Supplementary 1). Polymorphism of the candidate gene *Bo9g058630* was further confirmed in the segregating population.

**Table 4:** Primers used for full-length candidate gene cloning

Primers	Primers sequences (5’-3’)
Bo630	GTCCACAACACTTTCATACAA//TTCCCAAAGCATAATCCATCT

## Discussion

### Mapping of the purple leaf gene

Purple-leaf traits have been studied in several species, such as rice [30, 31], tetraploid wheat [32], *Brassica rapa* [7], and carrot [9, 33]. Some genes related to anthocyanins, which might be responsible for purple leaf color, have been reported in *Brassica*, including *BrPur* in *B. rapa* [7]*, BnaA.PL1* in *B. napus* [8], and the *Re* and *Pi* genes in *B. oleracea* [5, 9].

Ren et al. [5] reported that markers C9Z90 (C09:18,377,796 bp) and C9Z94 (C09:6,872,051 bp) are tightly linked to *Re*, which controls the red-leaf phenotype in kale [5]. The red-leaf phenotype is similar with the purple phenotype of the parents used in the present study. Based on reference genome ‘TO1000’, our results show that C9Z90 is located between InDel markers BRID517 (17,879,063 bp) and BRID530 (18,485,633 bp) and that C9Z94 is located between InDel markers BoID4704 (6,418,130 bp) and BoID4707 (7,177,017 bp). This indicates that the mapping interval in Ren *et al.* [5] is larger than ours. Furthermore, the distance between the *Re* gene and the closest markers are 2.0 cM for C9Z94 and 0.3 cM for C9Z90 [5] but 6.1 cM for BoID4704 and 1.1 cM for BRID517 in our mapping population, which may be caused by different mapping populations. In our study, however, the *BoPr* gene was successfully fine mapped to a 44.8-kb interval based in the ‘TO1000’ reference genome.

For the mapping population, F_2_ and BC_1_ populations are usually constructed for mapping important agronomic traits. In this study, for markers far away from the candidate gene, the recombination rate of the F_2_ population was larger than that of the BC_1_P_1_ population; however, the opposite case was true for markers close to the candidate region (Additional file 2: Table S1). The closest flanking markers BRID485 and BRID490 were all screened from the BC_1_P_1_ population, with no recombinants in the F_2_ population. Furthermore, the rate of recessive individuals in the BC_1_P_1_ populations was 1/2, which was twice that of the F_2_ population. Therefore, we used the large BC_1_P_1_ population to fine map the candidate gene.

### Possible assembly errors in cabbage reference genome ‘02–12’

With the development of technology, large amounts of data are produced by genome sequencing, though genome assemblies based on these data are often woefully incomplete. Although the Nipponbare reference sequence (RefSeq) exhibits the best quality among crop genomes, it still contains many assembly errors and gaps [34, 35]. Genome sequencing has been completed in three species of *Brassica*: *B.napus* [36], *B. rapa* [37] and *B. oleracea* [10, 11]. Some assembly problems have been reported. For example, Lee et al*.* [23] revised the ‘02–12’ reference genome assembly when they mapped clubroot resistance QTLs using genotyping-by-sequencing; new positions for 27 v-blocks and 10 s-blocks and several inversions of some alphabetically named blocks in reference genome ‘02–12’ and two mis-anchored sequence scaffolds in reference genome ‘TO1000’ were identified. In addition, Liu et al. [15] found possible assembly errors in ‘02–12’ while mapping a yellow-green leaf mutant.

In this study, the recombination rate of markers M17 and BoID4714 in ‘02–12’ [10] (48 kb/cM) was found to be almost 12 times higher than the usual level in the cabbage genome (~600 kb/cM) in preliminary mapping. In ‘TO1000’, the primary mapping interval was found to be 12.7 Mb (~1760 kb/cM). Based on the ‘TO1000’ genome, the *BoPr* gene was fine mapped to a 44.8 kb interval, but according to primary mapping, this 44.8 kb is not located in the 280 kb region but is on an unanchored scaffold (Scaffold000035_P2) of ‘02–12’. Thus, the mapping results suggested possible assembly error and the potential location of the unanchored Scaffold000035_P2 in the 02–12 reference genome. However, this scaffold is only 1.5 Mb. More unanchored scaffolds could be detected within this interval in the future if PacBio, a third-generation sequencing platform, is applied to improve the reference genome of ‘02–12’. These results will contribute to the improvement of the cabbage genome and provide guidance for map-based cloning of other genes in this region.

### The DFR gene in anthocyanin biosynthetic pathways

The biosynthetic pathways of anthocyanins and related genes have been well characterized, and DFR is a key enzyme in the anthocyanin biosynthetic pathway [38]. In characterizing DFR genes, Ahmed et al. found that these genes are associated with cold and freezing stresses in *B. rapa* [39]. Guo et al*.* identified 73 anthocyanin biosynthetic genes, locating the DFR gene on chromosome A09, named *BrDFR* (*Bra027457*), in *B. rapa* [40].

In our study, *Bo9g058630* was found to be homologous to *Arabidopsis thaliana* AT5G42800, which encodes DFR. DFRs utilizing the three primary dihydroflavonol substrates have been characterized. Expression of DFR clones together with plant-specific 4-coumaroyl: CoA ligase, chalcone synthase, chalcone isomerase, and flavanone 3-hydroxylase in *E. coli* resulted in the synthesis of various levels of pelargonidin and could be used for engineering of other bioactive flavonoids, such as flavan-3-ols [41]. The purple-leaf trait of ornamental kale is largely attributed to various anthocyanin components [2], and sequence alignment showed that the function of *Bo9g058630* in the white-leaf line W1835 is disrupted by two InDels. Furthermore, we designed a co-dominant marker based on sequence differences between W1835 and P1827, and this marker co-segregated with the phenotype of all recessive individuals in the F_2_ and BC_1_ populations. Thus, we suggest that *Bo9g058630* is the most likely candidate gene for the purple-leaf trait. However, further work involving transformation is needed to verify whether the function of this gene is responsible for purple leaves in kale.

## Conclusions

Inheritance of purple leaves in kale was found to be controlled by a single dominant gene, *BoPr*, and this gene was mapped to a 44.8 kb interval (reference genome ‘TO1000’) on chromosome C09. InDel markers BRID485 and BRID490 are closest to *BoPr*, flanking the gene at genetic distances of 0.1 cM and 0.2 cM. In the fine-mapping region, only *Bo9g058630* showed high homology to AT5G42800 (dihydroflavonol reductase), which was identified as a candidate gene for *BoPr*. This study lays a foundation for the cloning of the gene *BoPr* as well as further function analyses.

## Additional files

Additional file 1: Supplementary 1. Sequences of W1827 and P1835. (DOC 29 kb)
Additional file 2: Table S1. The recombination rate of markers far away from the candidate gene in the F_2_ and BC_1_P_1_ population. (DOCX 13 kb)

## Abbreviations

BRAD: Brassica Database
BSA: Bulked segregant analysis
InDel: Insertion/deletion
PCR: Polymerase chain reaction
QTL: Quantitative trait locus
SNP: Single nucleotide polymorphism
