# Supplementary material for: Genetics and fine mapping of a purple leaf gene, BoPr, in ornamental kale (Brassica oleracea L. var. acephala)
Source: BMC Genomics. 2017 Mar 14;18:230. doi: 10.1186/s12864-017-3613-x (PMC5348804; doi:10.1186/s12864-017-3613-x)
Supplement: Additional file 1: — Supplementary 1. Sequences of W1827 and P1835. (DOC 29 kb) [file 12864_2017_3613_MOESM1_ESM.doc]

**Title: Genetics and fine mapping of a purple leaf gene, *BoPr*, in** **ornamental kale (*Brassica oleracea* L. var. *acephala*)**

Journal name: BMC Genomics

Author names: Xiao-ping Liu, Bao-zhen Gao, Feng-qing Han, Zhi-yuan Fang, Li-mei Yang, Mu Zhuang, Hong-hao Lv, Yu-mei Liu, Zhan-sheng Li, Cheng-cheng Cai, Hai-longYu, Zhi-yuan Li, Yang-yong Zhang^*^

Affiliation: *Institute of Vegetables and Flowers, Chinese Academy of Agricultural Sciences, Beijing 100081,* People’s Republic of China

*e-mail:* [*Zhangyangyong@caas.cn*](mailto:Zhangyangyong@caas.cn)

**Supplementary information 1**

**Sequences of W1827**

ATGGTAGCTCACAAAGAGACCGTGTGCGTAACCGGCGCATCAGGATTCATTGGTTCATGGCTCGTGATCCACTTTGTCATAAGCTGTTCTAAGTATTTGCAGCTTCAATCTCGGATCTAAAACAGCCCCCATAGCTAGAACAAGACTGTATTCATCCCAATACTTAGAAAACTTAACTCTCATTTTCTTAGCCATCTCTTTCATTACTGGATCATCACAACTAGCATACTTCATCAGTAAGCATTCAATTTTCCACACTTGTAGAAAATATGCATTAGCCGTTGGATATCTAACGCCTGAGAAGAACGTTGTAATAGTGCTGAAAGGCTTCAGAAACTCACAAATTTTCTGCCCTCGATCCCACTCATCCTCTGAAGGCAATGATTTGTAACTCCTGTCACACTCTTTCAAACTAGTGAATGCGTCACGAAACTTCAGAGCTCTAGCAAGCATATCATAAGTTGAATTCCATCTGGTTGGTACATCTAGAGACAGTCCAGCTCCACTCCTAATCCCTACACTCTGAACACATGCTGCAAATGCTTCTATCCTTGATCCAGATGCTTTGACAAACTTAACACTCTCTCGGATATTTTCCAGAAGACCAACAGCAAGTTCTAAACCTTCTTTCACTATGAGATTCAAAATGTGTGCACAGCATCTAACGTGGAAGAACTTCCCATCACACAACAACCCGTTGCCGCTAGCCATTTGAAGTCGATGCTTGAGTATCTTCTGCATACTATCGTTATAAGTAGCATTATCCAAGGTCATAGAGAAGACCTTTTTCTCTAATCCCCACTCCTTCAAACAGCTAATAAGTTTGTTAGCCACTTCTTCACCGGTATGTGGAGGTTTCAACTCACTGAAGACTAGTATCTTGTTGTTCAACTTGAAGCTCTCATCAACATAGTGAGCTGTCAGACAGATATATCCCGTCATAGTAGTAGAAGCTGTCCATATATCTGAGGTAAAAGATACCCGACCCTTGAATTCTGCTAACTCTTTCTTCAGCTTCTCTCTTTCTTCCTCATATCTCTTATAGACATCAGCTCCAGCAGTTTGTCTAGATATATGCTGGCATTTGGGATTCAGGTACTTGTCCCTAGCTCTAACCTTCTCATACTCAACGTATTTGAAAGGCTGATCATGGAAAATAATGATCTCACTGATCATATCACGATCTACTTTCGGATCATACTCACGATCaACCACTTGACGGAGGCTTTTTGGGACAGATTTCTAAATGCCGTTTCATAGAAGATGTTCCTGAGGCAGATTCTGATACTAACTTCTTATTACAATGAATGCAACGACTCCTTCTTTTTCCATCTGCCTCTACTCCTACTACTACGAAATGGTTCCAAACAAGAGACTTTGCGCGTTTAGAATGACTCACAGTTTCAGTTGCTTGAACAGGTTGAGTTTCACTTTGTGCTTGTCCTTGAGTTTCTGCTTCATCATCATCATCAACCTCCATTTGCTCATTTGCAGCCTCAAGAGCTGCTAGTGTGTCAAGTGTTTGTGAATCCATCCTGGAAAAAACATTAAACAATCAGAAAATTTAAGCTATTATTGAATAGCGTAACGGCACACATACGCACACATAAAACAATATTATTGTTTCAGCACAAGACCAAACCACCACTGATAATCAACCTGTTAAGCACAACAACAAGAAAGCCCACAATATGCAAACGTAACGGCACACATGCGCACACATAGAACAATGTTGTTGTTAGCACAAAATCAATCAAACCCTGAGTTCACGACCTAAATTTTCTCTATAAATGGTTGATTAAACTTTTATTTTCTTTTTCTCTAACAAAACAAAGTACTTGATCGAACAAAGTGATTTTTTACAATCATTGTTGTCCGGGACAACTTGACCAACCAACCAATGATCTATAGCACAAAGACAAAACCCACTCAAGTTCTTCCAGAGAGTTTTAAGAACAAACAAAAGCATAGTTCTTACATTTTATAGAGAGAACGAAGAGGAAGGTAATCACGTACGGACTTCTTCTGGTAACACTTTGACTCCTTTCGTTGGGGCCTAAACCAATTAGGATAGAATCGATGGTTAGTCAACCAGATTCTAAAGCATGAGAATCAAAACACAAAAACACATTTCGAAATCTTACCAATGAACCCAATCTAGAATGAATCGTCGCAAGAAAGCATGTCTAATTTCTAGATCTGTGTTTTGTGGCTGTGGTTACGGGAGAAACAAAAATTTGGGGTAAAATATAATGTAAAACGACGTCGTTTCGGTTAATTTTTTTTTTAAAAAAAAAACCATCGGGTACCCGAAGCCCGATAGTGTAAACCCGATAGGGTAATAAACAAAACAAGACCCGCCCAACAAAAACCCGCGAGTTTTAAAATCTAAAAGTACGGGTTTCGGGTTTCAAATTTCAACCGGGCTTCGGGTACCCTATGGGTAAAAACCCATTATTAACATCCCTACTTACGAGTTTTCTTGGCCTGTAAAGGAAATTTGAAGAAAGTGCAACATCTTCTTGATTTGCCAAACGCGAAGACGCAACTCACTTTATGGAAAGCCGATTTATCTGACGAAGGAAGCTACGATGACGCCATAAACGGATGCGACGGCGTTTTTCACATAGCTACTCCCATGGATTTTGAATCCAAGGATCCCGAGGTGAGTTATACTATGAACCTTTTTCTTATTACACATCAATCCTACAAGATTTTGTTAAATGAGTTTGTTTGAATCAGAACGAAGTGATAAAACCAACAGTGAATGGAGTGTTGGGGATAATGAAAGCATGTGATAAGGCAAAGACCGTACGAAGAATTGTGTTTACTTCGTCTGCTGGAACGGTTAATGTtGAGgAACACCAgAAAAATGTCTATGATGAAAACGATTGGAGTGATCTCGACTTTATCATGTCCAAGAAGATGACAGGATGGGTATATATATTAAGGATCATATATAAAAAATTAACCCGAGGTTGATCTTCTTCAAAGTAATTTATGTTTTTGATAAATTGTTGGCAGATGTATTTCATGTCGAAAACGTTAGCCGAGAAAGCAGCTTGGGATTACGCTAAGGAAAAAGGAATAGATTTCATTAGTATTATCCCGACATTGGTGATCGGTCCATTTATAACAACATCTATGCCGCCTAGCCTTATTACCGCGCTCTCTCCTATCACTCGTGAGTGAGCCTACTTTCTAATCCCTCTTTTTTAACTAAGAGGTTAATTTAAAACGGTAAAAATGTTTTAGGTAACGAGGCACATTACTCCATCATAAGACAAGGACAGTATGTCCACTTGGACGACTTATGCAATGCTCATATATTCTTGTACGAACAAGCTGCTGCCAAGGGACGTTATGTTTGTTCCTCTCACGATGCAACGATTCTTACTATCTCCGAGTTTCTCAGGCAAAAATATCCAGAATATAACGTGCCTTCAACGTAAGATTTTTATCATTACCGGTTTAAGCTTTTTTTCCATATTCAGTTTAATTTTTTTTTTTCTGAATATGAACTCTTTGGAACAGGTTTGAAGGAGTGGATGAGAATCTAAAGAGCATTATGTTCAGTTCCAAGAAGCTGATTGATATGGGATTTAACTTCAAGTATAGTCTCGAGGATATGTTGGTGGAATCGATTGAGACATGTCGTCAAAAGGGTTTTCTCCCTGTCACTTTACCGGAACATTTGAAATCTGAGGACAAAGTTCCGGGCAGTGATGACAATAAGGAGATTAAAAACGGATCTGCAGGTTTAACTGATGGTATGGTAGCTTGTAAGAAGACCGAACCAGGGATGGCCGGCGAGAAAGCCGATAGTCACATGTCGGCACAGCAGATCTGTGCTTAG

**Sequences of P1835**

ATGGTAGCTCACAAAGAGACCGTGTGCGTAACCGGCGCATCAGGATTCATTGGTTCATGGCTCGTGATGCGGCTACTGGAACGTGGTTACTTTGTCCGTGCCACTGTTCGCGATCCTGGTACGTATCTTACAAACTCGTTAATTTCTCCTAAGAGTATATGTTAATACGTATCACTTTGTGTGTTTTAAGTAACTTACGAGTTTTCTTGGCCTGTAAAGGAAATTTGAAGAAAGTGCAACATCTTCTTGATTTGCCAAACGCGAAGACGCAACTCACTTTATGGAAAGCCGATTTATCTGACGAAGGAAGCTACGATGACGCCATAAACGGATGCGACGGCGTTTTTCACATAGCTACTCCCATGGATTTTGAATCCAAGGATCCCGAGGTGAGTTATACTATGAACCTTTTTCTTATTACATATCAATCCTACAAGATTTTGTTAAATGAGTTTGTTTGAATCAGAACGAAGTGATAAAACCAACAGTGAATGGAGTGTTGGGGATAATGAAAGCATGTGATAAGGCAAAGACCGTACGAAGAATTGTGTTTACTTCGTCTGCTGGAACGGTTAATGTTGAGGAACACCAGAAAAATGTCTATGATGAAAACGATTGGAGTGATCTTGACTTTATCATGTCCAAGAAGATGACAGGATGGGTATATATATTAAGGATCATATATAAAAAATTAACCCGAGGTTGATCTTCTTCAAAGTAATTTATGTTTTTGATAAATTGTTGGCAGATGTATTTCATGTCGAAAACGTTAGCCGAGAAAGCAGCTTGGGATTACGCTAAGGAAAAAGGAATAGATTTCATTAGTATTATCCCGACATTGGTGATCGGTCCATTTATAACAACATCTATGCCGCCTAGCCTTATTACCGCGCTCTCTCCTATCACTCGTGAGTGAGCCTACTTTCTAATCCCTCTTTTTTAACTAAGAGGTTAATTTAAAACGGTAAAAATGTTTTAGGTAACGAGGCACATTACTCCATCATAAGACAAGGACAGTATGTCCACTTGGACGACTTATGCAATGCTCATATATTCTTGTACGAACAAGCTGCTGCCAAGGGACGTTATGTTTGTTCCTCTCACGATGCAACGATTCATACTATCTCCGAGTTTCTCAGGCAAAAATATCCAGAATATAACGTGCCTTCAACGTAAGATTTTTATCATTACCGGTTTAAGCTTTTTTTGCATATTCAGTTTAATTTTTTTTTTtCTGAATATGAACTCTTTGGAACAGGTTTGAAGGAGTGGATGAGAATCTAAAGAGCATTATGTTCAGTTCCAAGAAGCTGATTGATATGGGATTTAACTTCAAGTATAGTCTCGAGGATATGTTGGTGGAATCGATTGAGACATGTCGTCAAAAGGGTTTTCTCCCTGTCACTTTACCGGAACATTTGAAATCTGAGGACAAAGTTCCGGGCAGTGATGACAATAAGGAGATTAAAAACGGATCTGCAGGTTTAACTGATGGTATGGTAGCTTGTAAGAAGACCGAACCAGGGATGGCCGGCGAGAAAGCCGATAGTCACATGTCGGCACAGCAGATCTGTGCTTAG
