# Supplementary material for: Genetics and fine mapping of a purple leaf gene, BoPr, in ornamental kale (Brassica oleracea L. var. acephala)
Source: BMC Genomics. 2017 Mar 14;18:230. doi: 10.1186/s12864-017-3613-x (PMC5348804; doi:10.1186/s12864-017-3613-x)
Supplement: Additional file 2: Table S1. — The recombination rate of markers far away from the candidate gene in the F2 and BC1P1 population. (DOCX 13 kb) [file 12864_2017_3613_MOESM2_ESM.docx]

**Table S1**

| Primers | No. of recombinants in F_2_ population | No. of white leaf individuals | genetic distance  (cM) | No. of recombinants in BC_1_P_1_ population | No. of white leaf individuals | genetic distance  (cM) |
| --- | --- | --- | --- | --- | --- | --- |
| BoID4707 | 60 | 338 | 17.7 | 50 | 991 | 5 |
| BRID471 | 5 | 338 | 1.4 | 5 | 991 | 0.5 |
| BRID472 | 3 | 338 | 0.8 | 5 | 991 | 0.5 |
| BRID482 | 2 | 338 | 0.6 | 2 | 991 | 0.2 |
| BRID485 | 0 | 338 | - | 1 | 991 | 0.1 |
| BRID490 | 0 | 338 | - | 2 | 991 | 0.2 |
| BRID494 | 0 | 338 | - | 3 | 991 | 0.3 |
| BRID499 | 6 | 338 | 1.7 | 7 | 991 | 0.7 |
| BRID517 | 9 | 338 | 2.6 | 14 | 991 | 1.4 |
| BRID530 | 14 | 338 | 4.1 | 14 | 991 | 1.4 |
